# CUMULATIVE DISADVANTAGE OF EARLY-LIFE ADVERSITY AND HEALTH IN MIDLIFE AND LATER ADULTHOOD

**DOI:** 10.1093/geroni/igz038.1270

**Published:** 2019-11-08

**Authors:** Jooyoung Kong, Agus Surachman, Deborah Carr

**Affiliations:** 1 University of Wisconsin-Madison, Madison, United States; 2 The Pennsylvania State University, University Park, Pennsylvania, United States; 3 Boston University, Boston, Massachusetts, United States

## Abstract

Cumulative dis/advantage (CDA) framework is one of the most influential theoretical frameworks in understanding how early adversity creates health disparities across adulthood. The CDA model posits that adverse experiences early in life may lead to subsequent adversities over time and accumulates across the life course. Various studies have shown that middle-aged and later adulthood are periods when accumulated disadvantages proliferate, resulting in heightened risks for an individual’s health and well-being. This symposium includes four presentations that build on such existing knowledge, and its primary aim was to further examine the complexity of how various types of adverse childhood experiences may influence physical and psychological health in middle and later adulthood. This symposium addresses a wide range of early adversities, including low socioeconomic status, parental maltreatment, and household dysfunctions. The four presentations also focus on examining various aspects of physical and psychological health outcomes in later adulthood, including measures of body mass index, physical functional ability, somatic symptoms, and clinical risk for rapid declines in kidney function. Furthermore, these presentations will demonstrate the utilization of innovative and robust methodological approaches, including latent class analysis, multilevel structural equation modeling, and latent growth modeling on examining the association between early life adversity on the long-term trajectory of change in health status using large-scale longitudinal data. Lastly, this symposium consists of an outstanding group of multidisciplinary presenters with diverse backgrounds who aim to enhance the understanding of the processes and mechanisms of CDA and how they affect individuals’ life courses.

